# Magnetically stimulated transient creep processes in a heterogeneous aluminum alloy with ferromagnetic inclusions

**DOI:** 10.1038/s41598-025-16116-z

**Published:** 2025-08-20

**Authors:** Vladimir Nikolaev, Mounir Friha, Arkady A. Skvortsov, Danila Pshonkin, Aleksandr Abramov, Polina Kuznetsova, Alexandr Kazak

**Affiliations:** 1 Moscow Polytechnic University, Moscow, Russia; 2 Department of Dynamics and Strength of Machines and Materials, Moscow Polytechnic University, Moscow, Russia

**Keywords:** Aluminum alloy, Iron-based ferromagnetic inclusions, Creep, Transient processes, Elastic moduli, relaxation time, Electrical and electronic engineering, Physics

## Abstract

This study analyzes the creep processes of a heterogeneous aluminum alloy containing ferromagnetic inclusions with an average size of 3–5 μm, and the influence of preliminary magnetic exposure (in a constant magnetic field (MF) with induction B < 0.75 T) on these processes. Experimental investigations were carried out to determine the characteristic times of the transient stages of short-term creep, which do not exceed approximately 25 ms. We analyzed the transient deformation behavior of the aluminum-based alloy and consequently determined the elastic moduli of the material, along with the influence of preliminary magnetic exposure on them. It was established that the MF has the most pronounced effect on the “long-term” elastic modulus *H* (defined as the ratio of stress to the relative strain of the material after sustained application of a constant load). We propose that the observed linear decrease in *H* with increasing *B* is associated with the magnetostriction of the inclusions during preliminary magnetic exposure. An increase in magnetic induction enhances local stresses at the matrix–inclusion interfaces, which in turn leads to a rise in dislocation density. These microstructural changes influence the subsequent deformation behavior, including both transient responses under loading and stress relaxation during unloading. We conclude that the elastic modulus *H* is the most sensitive parameter to the influence of magnetic fields, indicating a significant impact of external MF on the creep dynamics of the structurally heterogeneous aluminum alloy under investigation.

## Introduction

Creep is the tendency of a material to undergo continuous deformation under the influence of a constant load. Most solids are susceptible to creep to some extent^1,2^. In single crystals, creep mechanisms are generally associated with dislocation dynamics, the diffusion of point defects, and possible microstructural evolution^3,4^. Numerous studies have been devoted to creep in single-phase materials, providing valuable insights into the physical mechanisms underlying the process^1,5,6^.

The study of creep in polycrystalline materials is equally important. In this context, classical diffusion creep models are commonly applied, including Nabarro–Herring creep, based on bulk diffusion, and Coble creep, governed by grain boundary diffusion^7^. For instance, Ankem et al.^8^ and McLean et al.^9^, based on extensive experimental data, concluded that the mechanical behavior of two-phase alloys is strongly influenced by the individual phases’ mechanical properties, volume fractions, and morphologies. In general, the creep behavior of two-phase alloys cannot be accurately predicted using simple mixing laws, due to significant interphase interactions. Moreover, when analyzing elastoplastic deformation, Ankem et al.^8^ emphasized the crucial role of differences in the elastic properties of constituent phases in initiating plastic flow. For example, a phase with lower critical stress (for slip or twinning) may undergo plastic deformation at stress levels below its intrinsic threshold due to interfacial elastic effects.

In the context of creep (*T* < 0.25 T_liq_, where T_liq_ is the melting point) in two-phase alloys, it has been observed that such materials can deform via mechanisms distinct from those operating in their individual phases. The actual creep mechanisms and the extent of deformation depend on the morphology and crystallographic orientation of the constituent phases.

It is also noteworthy that in such systems, the mechanisms of creep (i.e., slow plastic deformation under constant loads) become more complex due to structural heterogeneities, which lead to increased local mechanical stresses and nonlinear interactions between the metal matrix and inclusions. It is well established that the presence of inclusions creates additional barriers to dislocation motion, resulting in increased dislocation density at interphase boundaries, the formation of zones with differing deformation kinetics, and the emergence of complex stress redistribution patterns^1,2,5^. In these systems, the steady-state creep stage is often governed by dislocation climb, whose activation energy is close to that of self-diffusion. However, transient creep regimes (unsteady-state creep) are typically characterized by the interplay between strain hardening and dynamic dislocation recovery^7,9^.

To date, no comprehensive model exists that accounts for the interrelation between morphology, phase orientation, and deformation mechanisms, and reliably predicts the creep process of various two-phase alloys.

For instance, on one hand, microinclusions can promote a more uniform strain distribution, but on the other hand, they can reduce fatigue resistance. In another example, numerical models developed for several aluminum and titanium alloys successfully describe transient creep under multiaxial loading, yet they often neglect the influence of phase morphology and crystallographic orientation. The deformation mechanisms in two-phase systems—such as slip, twinning, and martensitic transformations—vary significantly depending on alloy composition and heat treatment, further complicating the development of unified predictive models. Consequently, modern modeling approaches remain fragmented: some focus primarily on dislocation kinetics, others emphasize environmental effects, while still others address phase stability. The absence of a unified framework that integrates morphology, phase orientation, and multimodal deformation mechanisms remains a major challenge in predicting the creep process of structurally heterogeneous alloys.

As for aluminum alloys, the presence of inclusions and structural heterogeneity also exerts a significant influence on transient creep process. This explains why many researchers have conducted experimental investigations aimed at accumulating a broader set of data on heterogeneous aluminum-based alloys. For instance, it was experimentally demonstrated that the yield strength of an Al–Li alloy varied from 378 to 456 MPa depending on the rolling direction, within an angular range of 0° to 90°. Anisotropic deformation behavior under creep conditions was also reported^10^. In a study of aerospace-grade aluminum alloys—specifically the Al–Cu–Li 2050 alloy—it was found that the material contained the Al_2_Cu phase at low Li concentrations (< 0.6%), while the dominant strengthening phase shifted to Al_2_CuLi when the lithium content increased to 1.4–1.5%. The authors emphasized the decisive role of this phase in the alloy’s strengthening behavior, a process notably enhanced by the addition of Cu^10,11^. In the case of structurally heterogeneous Al–Zn–Mg alloys, both microstructural transformations and changes in macroscopic mechanical properties were observed as a function of non-isothermal creep aging, carried out under varying thermal conditions^12^.

A study on the aluminum alloy Al–Cu–Ag–Mg demonstrated a reduction in creep strain^13^, attributed to the presence of microinclusions (1–2 μm) that effectively acted as dislocation pinning centers. The alloy’s microstructure, containing Ω (Al–Cu–Ag–Mg) and Θ′ (Al–Cu) precipitates, was found to be stable and exhibited excellent mechanical performance at 200 °C. As a result, the alloy demonstrated enhanced creep resistance, with minimum strain rates on the order of 10^−8^–10⁻^6^ s⁻^1^ at 200 °C under applied mechanical stress ranging from 120 to 180 MPa^13^.

Thus, the presence of inclusions in metallic samples significantly alters the creep process. Consequently, numerous researchers have investigated metal matrix composites (MMCs) based on aluminum. For example, Gupta et al.^14^ examined the creep process of aluminum matrix composites reinforced with Al_3_Ti particles (with varying mass fractions up to 8 wt%) under applied stresses ranging from 113 to 170 MPa and temperatures between 543 and 603 K. Microstructural analysis revealed a uniform distribution of Al_3_Ti particles (average size ~ 1 μm) throughout the matrix, facilitated by ultrasonic mixing. The findings indicated that creep process in the Al_3_Ti composite was primarily governed by diffusion-controlled dislocation motion, consistent with previously reported creep mechanisms in aluminum-based metal matrix composites^6,15^.

An interesting area of research involves studying the behavior of aluminum alloys of the Al–Fe–Cu type. For instance, Chen et al.^16^ investigated the creep process of 8030 aluminum alloys under compressive loading at deformation temperatures of 200–250 °C and applied stresses of 20–40 MPa. In the initial-state samples, spherical inclusions (~ 0.2 μm in diameter) and loop dislocations were observed near subgrain boundaries, forming heterogeneously along grain and subgrain boundaries. According to Chen et al.^16^, the particles distributed along these interfaces were predominantly Al_3_Fe, which not only inhibited grain boundary migration but also suppressed subgrain growth, thereby enhancing the creep resistance of the alloy. Furthermore, it was found that at 458 K, the inclusions increased in size, resulting in substantial changes in creep characteristics^16^. As previously established, iron-containing aluminum alloys exhibit sensitivity to external magnetic fields (MFs). In this context, Skvortsov et al.^17^ examined the effect of constant MFs (with magnetic induction *B* = 0.7 T) on the creep process of such alloys. They demonstrated that preliminary exposure of samples (with ≈ 0.4 at% Fe) to a constant MF (*B* = 0.7 T for 30 min at room temperature) led to an increase in total creep strain of up to 35%^17^.

To clarify both experimental and theoretical aspects of creep, investigations are typically carried out using simplified combinations of hardening mechanisms under conditions in which the morphological features of the microstructure (such as grain size and precipitates) remain stable during mechanical testing.

As previously noted, in most cases, creep process in metals and alloys is governed by dislocation dynamics during plastic deformation^6,13–18^. The characteristic times of transient creep under stepwise loading conditions can vary significantly depending on the material, applied mechanical stress, temperature, and other factors—ranging from milliseconds or seconds (short-term creep)^19,20^ to several days, months, or even years (long-term creep)^8,9,21^. For example, a study of Al–Mg alloys using acoustic emission techniques revealed that primary deformation processes initiated within a few milliseconds^20^. In contrast, creep in Al–Li alloy samples subjected to uniaxial tension developed only after several hours^21^. Characteristic deformation times as long as 18 h were observed when electric pulse processing was used to enhance the creep process of Al–Li alloys^8,9^.

Therefore, creep process in structurally heterogeneous alloys (including aluminum-based ones) is strongly influenced by the presence of inclusions, which act as additional stress concentrators, serve as sites for the localization of structural defects, and determine the characteristic timescales of transient creep. Aluminum alloys with iron-containing inclusions are particularly noteworthy due to their (1) potential sensitivity to external magnetic fields (MFs) and (2) ability to significantly alter the mechanical response of the alloy. However, there is a lack of comprehensive studies on the creep process of structurally heterogeneous aluminum-based alloys. In particular, transient responses during stepwise loading/unloading under creep conditions, and the influence of preliminary magnetic exposure on these processes, remain insufficiently explored. These issues are important not only for advancing the understanding of structure-sensitive properties in heterogeneous materials but also for the design and development of smart materials with tunable mechanical characteristics.

Accordingly, the aim of this study was twofold: (1) to investigate the transient creep process of an aluminum alloy containing iron-based inclusions under stepwise loading/unloading, and (2) to evaluate the influence of prior exposure to a constant magnetic field on these processes.

## Methodology

**Fig. 1:**
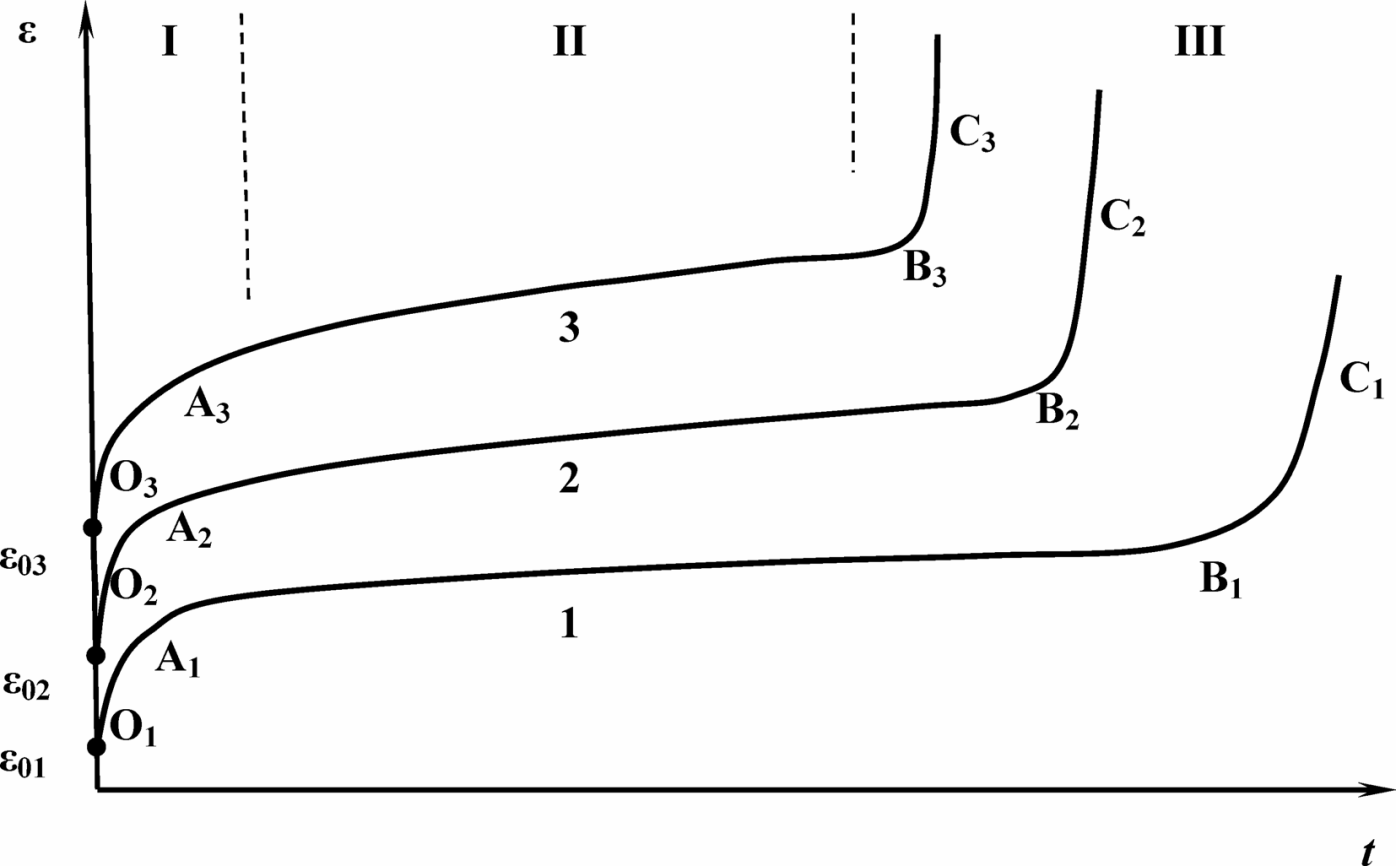
Family of creep curves with various magnitudes of tensile stresses: σ_1_ < σ_2_ < σ_3_. OA ― creep stage I (transition process); AB ― creep stage II (steady creep mode); B**C** ― stage III (high strain rate mode, ends either with a brittle fracture near point B, or with ductile fracture with formation of a neck).

**Fig. 2:**
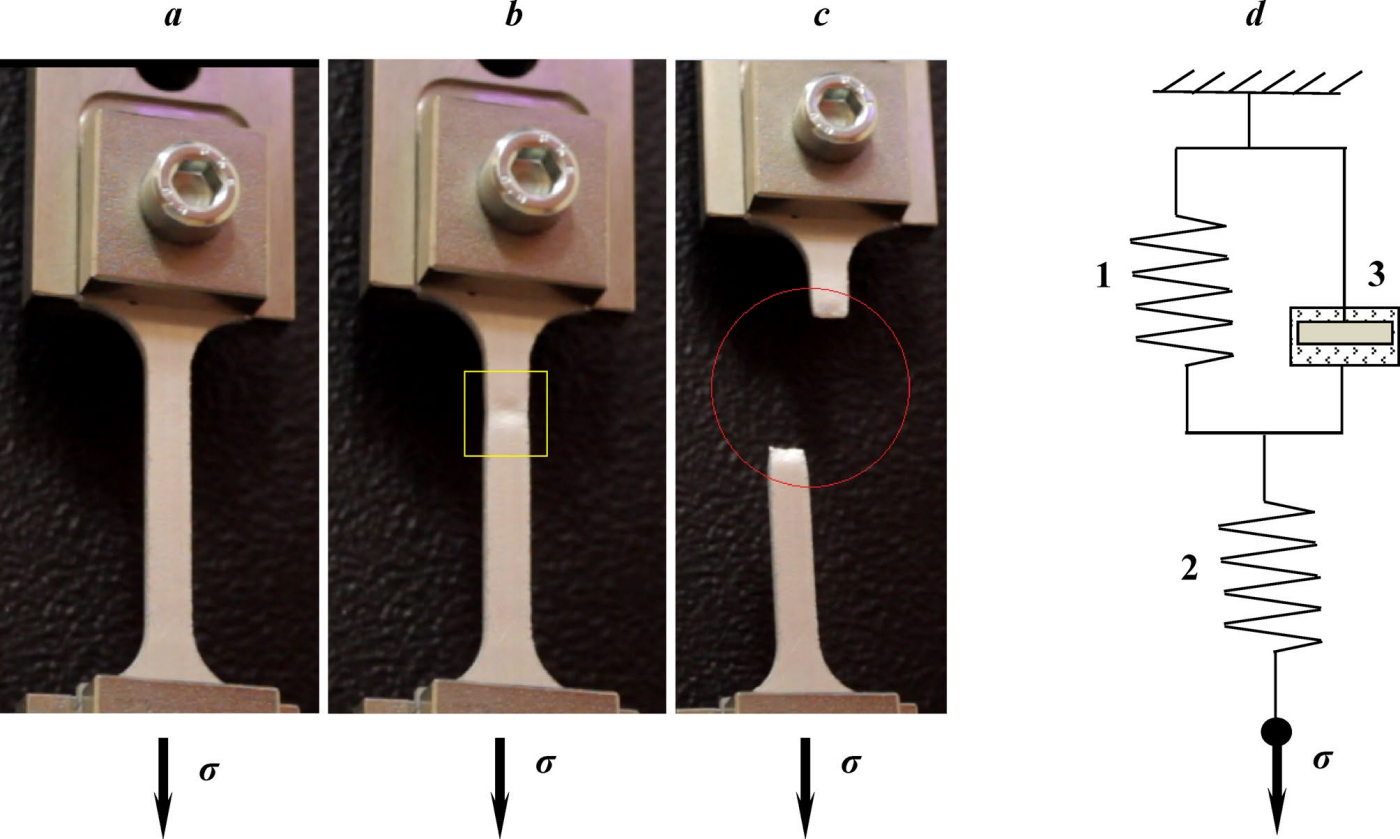
Sample deformation under creep conditions at stages I and II (***a***) and stage III (***b***,*** c***). The Kelvin viscoelastic model (***d***).

In materials science, creep (also referred to as cold flow) describes the ability of a solid to undergo slow, time-dependent deformation under constant mechanical stress. Typically, this occurs under prolonged exposure to stress levels below the material’s yield strength. To investigate material creep, long-term tensile or compression tests are conducted on samples under various stresses and temperatures. The resulting data are used to plot creep curves, i.e., the dependence of strain on time (Fig. 1). The onset of Stage III (Fig. 1) generally precedes failure (Fig. 2). Because the transition to Stage III is commonly deemed unacceptable for engineering applications, it is excluded from creep design considerations. Accordingly, the present study focused on Stages I and II under short-term creep conditions.

Following previous studies^22–24^, flat aluminum samples^1^ (Chemical composition of the aluminum alloy according to EDS analysis is C_Al_ = 96 ± 1%; C_Fe_ = 3 ± 1.5% and the concentration of residual impurities C_Mg_, C_Si_, Cu, Ti, Zn is approximately 1%). The presence of iron in the molten aluminum was ensured by the contact of the steel-smelting furnace with molten aluminum. Long-term persistence of aluminum in the furnace results in a 2% concentration of Fe in Al at a temperature of 700 °C (the eutectic in the Al-Fe system is formed at the 1.7% iron content at a temperature of 655 °C). At a temperature of 800 °C, the percentage of iron reaches 5%.) were used, consisting of (1) gripping heads for fixture within the testing machine (Fig. 2), and (2) a gauge section with constant cross-sectional area *S*_*0*_ *= h*_*0*_ × *b*_*0*_ (*h*_*0*_ = 2 mm, *b*_*0*_ = 5 mm) and a length *ℓ*_*0*_ of 30 mm. A 5-mm-long transition region between the heads and the gauge section was introduced to reduce mechanical stress concentrations.

Creep under uniaxial loading was studied using a lever-type system (WP-600 Creep Testing Machine, Germany) with a force ratio of 1:10, which allowed for (1) application of a constant load during testing, (2) smooth application and removal of load, and (3) loading up to a maximum force of 200 N. Measurements were conducted at room temperature. Sample elongation Δℓ was recorded using a high-precision micrometer (MICRON, Russia; resolution ± 10 μm). The testing system also included a digital camera (Canon 600D, Japan) to capture the deformation and fracture process. Additionally, it was used to record readings from the pointer strain indicator of the WP-600 machine at 30 frames per second, allowing for accurate reconstruction of the non-stationary stage of the creep process. The error in time measurement was ± 10 s. Each data point was obtained from processing the results of at least five samples, ensuring reproducibility and statistical reliability.

A constant MF was generated using neodymium magnets, with maximum MF induction *B* = 0.8 T. The MF induction in the gap could vary in the range *B* = 0.2–0.8 T and was measured using a digital Gauss meter (ZMST-5, India; operating range: 0–2.4 T). Following the protocols of prior studies^22,24^, the preliminary exposure time (i.e., before mechanical loading) in the MF was 30 min at room temperature.

**Fig. 3:**
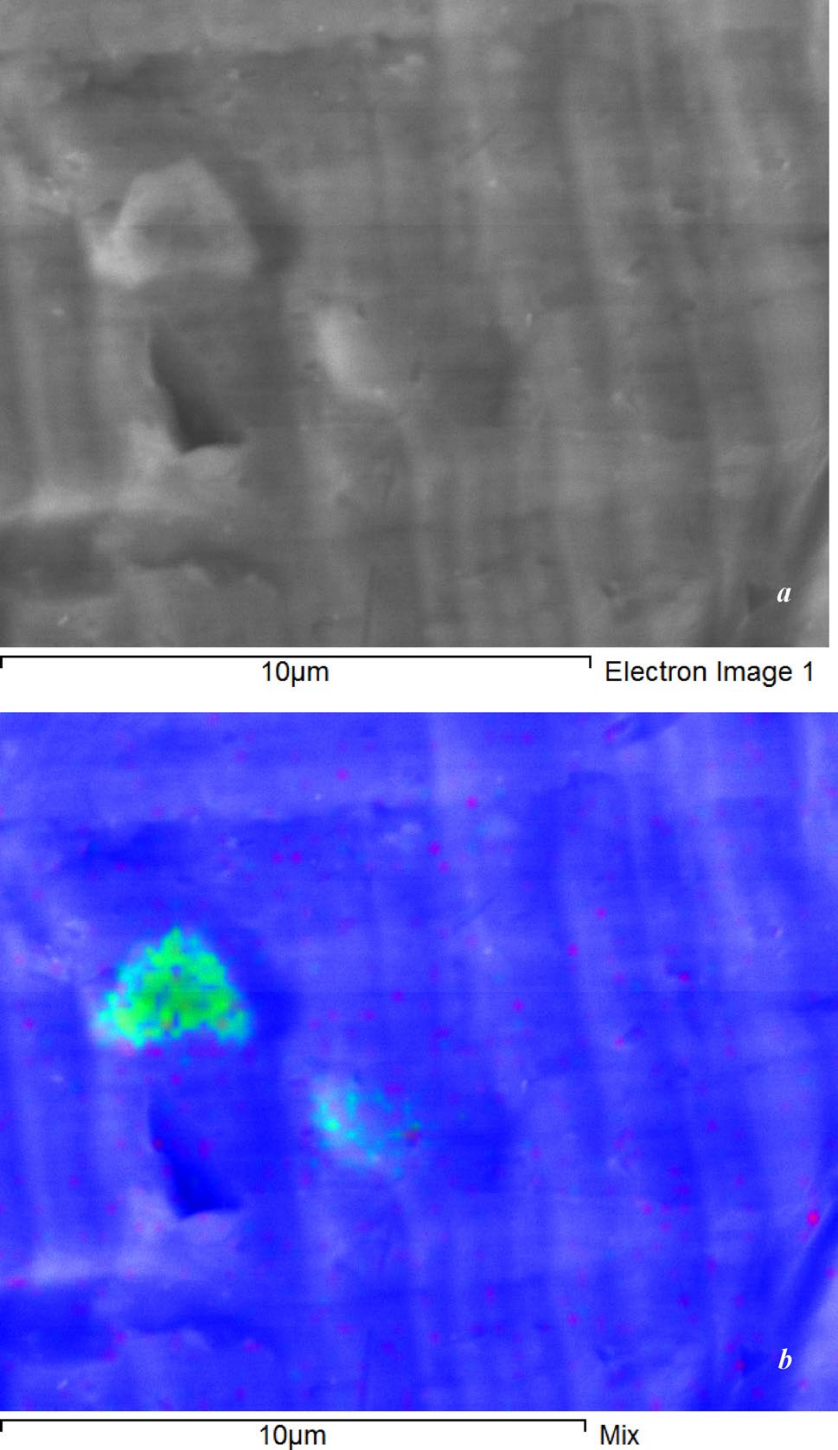
Scanning electron microscopy image of the sample surface (*a*) with color distribution of element concentrations (*b*) on the section surface: O (red) + Fe (green) + Al (blue).

After MF exposure, samples were subjected to creep tests. The microstructure and chemical composition of the matrix and inclusions were analyzed using a high-resolution field-emission scanning electron microscope JSM-7500 FA (JEOL, Japan) and an optical microscope Metam R-1 (LOMO, Russia). The average size of the inclusions was approximately 3 μm (Fig. 3).

The results of our microscopic studies and those obtained earlier^22,24^ confirm that iron in aluminum is present in the form of inclusions. Previously, the dependence of magnetic moment of a sample, *M*, on MF was obtained in the form of magnetic hysteresis, which identified the presence of ferromagnetic inclusions consisting of the well-known family of Fe_x_Al_1−x_ alloys (at x = 86–87%)^22,24^. These materials are known for high magnetostriction^25^.

Estimation of mechanical stresses σ_m_ normal to the inclusion–matrix interface, conducted according to the relation σ_m_ = λ_m_*B* (where λ_m_ = 8 × 10^8^ N/(T × m^2^) represents the proportionality coefficient (characterizes the material magnetomechanical sensitivity) of the Fe_x_Al_1−x_ alloy^25,26^, enabled to determine the normal stress σ_m_ to be 560 MPa when the sample was exposed to an MF with an induction *B* of 0.7 T. The resulting stresses σ_m_ exceeded the yield strength of aluminum (σ_Y_ ~ 200 MPa) and were sufficient to form a zone of freshly introduced dislocations around the inclusion after the sample’s exposure to the MF. These “activated” zones contributed to changes in macroscopic mechanical characteristics of the samples^9,21^, including those at the final stages of the “neck” formation and their destruction^22,24^. It is important to emphasize that in this study, mechanical stress increased linearly with magnetic field induction up to *B* = 0.7 T. The applied values of *B* in our experiments were lower than the saturation fields for the alloys studied, which exceed 1 T at room temperature^26^. Therefore, an increase in magnetic field induction leads to an increase in the magnetostriction magnitude in our entire range of *B* (0‒0.7 T).

Furthermore, we considered transient processes at stages I and II of creep of an aluminum alloy with iron-containing inclusions before and after exposure of the samples to a constant MF under conditions of stepwise loading/unloading of the samples.

To study transient processes during loading/unloading of the samples under creep conditions, we used a viscoelastic Kelvin body^22,27,28^ and the equation relating the effective mechanical stress *σ* and the relative deformation of the sample *ε*:

1 $$\sigma +n\frac{{d\sigma }}{{dt}}=H\varepsilon +nE\frac{{d\varepsilon }}{{dt}},$$

where *n* represents relaxation time.

In the case of extremely rapid application of the load, when $n\frac{{d\sigma }}{{dt}}>>\sigma$ and $nE\frac{{d\varepsilon }}{{dt}}>>H\varepsilon$, the first terms on the right and left sides of Eq. (1) can be neglected^22,27^. Accordingly, the equation takes the following form:

2 $$\frac{{d\sigma }}{{dt}}=E\frac{{d\varepsilon }}{{dt}},\:\text{and}\:\text{therefore}\:\sigma =E\varepsilon.$$

Consequently, the value *E* refers to the elastic modulus (i.e., the ratio of stress to relative deformation) during deformation at the moment of loading as rapid deformation. In the case of extremely slow load application, when the derivatives in Eq. (1) are small and can be neglected, then we have the following:

3 $$\sigma =H\varepsilon,$$

where *H* represents at this instance “long-term” modulus of elasticity (i.e., the ratio of stress to relative deformation of the material after long-term application of constant load)^22^.

Let us assume that the straight rod is stretched by a force *F* that does not change with time, i.e., *σ* = *σ*_0_ = const. At *t* = *t*_*1*_ = 0, the initial deformation of the rod is *σ*(0) = *σ*_0_ = const. The initial deformation of the rod at time *t*_*1*_ = 0 is then *ε(0)* = *ε*_*0*_ = *σ*_*0*_/*E*_*0*_. Let us determine the change in deformation with time. Because the voltage is constant, $\frac{{d\sigma }}{{dt}}=0$ and Eq. (1) takes the following form:

4 $${\sigma _0}=H\varepsilon +nE\frac{{d\varepsilon }}{{dt}} \:\:\:\:\:\:\:\text{or}$$

5 $$\frac{{d\varepsilon }}{{dt}}+\frac{H}{{nE}}\varepsilon =\frac{{{\sigma _0}}}{{nE}}.$$

We express the general solution of the heterogeneous Eq. (5) as the sum of the general solution of the homogeneous equation *ε*^0^ and the particular solution of the heterogeneous equation *ε*^00^:

6 $$\varepsilon ={\varepsilon ^0}+{\varepsilon ^{00}}$$

Assuming *ε*^00^ = const, from Eq. (5), we immediately determine ${\varepsilon ^{00}}=\frac{{{\sigma _0}}}{H}$. Solution of the homogeneous Eq. 

7 $$\frac{{d{\varepsilon ^0}}}{{dt}}+\frac{H}{{nE}}{\varepsilon ^0}=0$$

is sought in the form *ε*^*0*^*(t)* = *Сexp(kt*). Equation (7) then takes the following form:

8 $$k\exp (kt)+\frac{H}{{nE}}\exp (kt)=0.\quad \text{and} \quad k= - \frac{H}{{nE}},$$

and the following is the solution to the equation:

9 $${\varepsilon ^0}(t)={C_1}\exp \left( { - \frac{H}{{nE}}} \right).$$

Thus, the following is the solution to the inhomogeneous Eq. (7):

10 $$\varepsilon (t)=\frac{{{\sigma _0}}}{H}+{C_1}\exp \left( { - \frac{H}{{nE}}} \right)$$

We determine the constant *C* from the initial conditions as $\varepsilon (0)=\frac{{{\sigma _0}}}{E}$. We then have the following:

11 $${C_1}= - {\sigma _0}\left( {\frac{1}{H} - \frac{1}{E}} \right).$$

The final solution takes the following form:

12 $$\varepsilon (t)=\frac{{{\sigma _0}}}{H}\left( {1 - \left( {1 - \frac{H}{E}} \right)\exp \left( { - \frac{H}{{nE}}} \right)} \right).$$

If unloading occurs at time *t* = *t*_*2*_, i.e., *σ*(*t*_*2*_) = 0, then the rod deformation instantly decreases by the amount *ε*_*0*_ = *σ*_*0*_/*E* and becomes ${\varepsilon _{00}}=\varepsilon ({t_2}) - {\varepsilon _0}$. Let us now consider the dynamics of *ε* after unloading. In this case, the behavior of the deformation *ε*(*t*) can be determined using the following homogeneous equation:

13 $$\frac{{d\varepsilon }}{{dt}}+\frac{H}{{nE}}\varepsilon =0.$$

The following is the solution to Eq. (13):

14 $$\varepsilon (t)={C_2}\exp \left( { - \frac{H}{{nE}}t} \right).$$

We determine the integration constant under the condition that the deformation is $\varepsilon ={\varepsilon _{00}}$ at *t* = *t*_*02*_:

15 $${\varepsilon _{00}}={C_2}\exp \left( { - \frac{H}{{nE}}{t_2}} \right).$$

Hence,

16 $${C_2}={\varepsilon _{00}}\exp \left( {\frac{H}{{nE}}{t_2}} \right).$$

Thus, we obtain the following for the case of stepwise unloading:

17 $$\varepsilon (t)={\varepsilon _{00}}\exp \left( { - \frac{H}{{nE}}(t - {t_2})} \right).$$

**Fig. 4:**
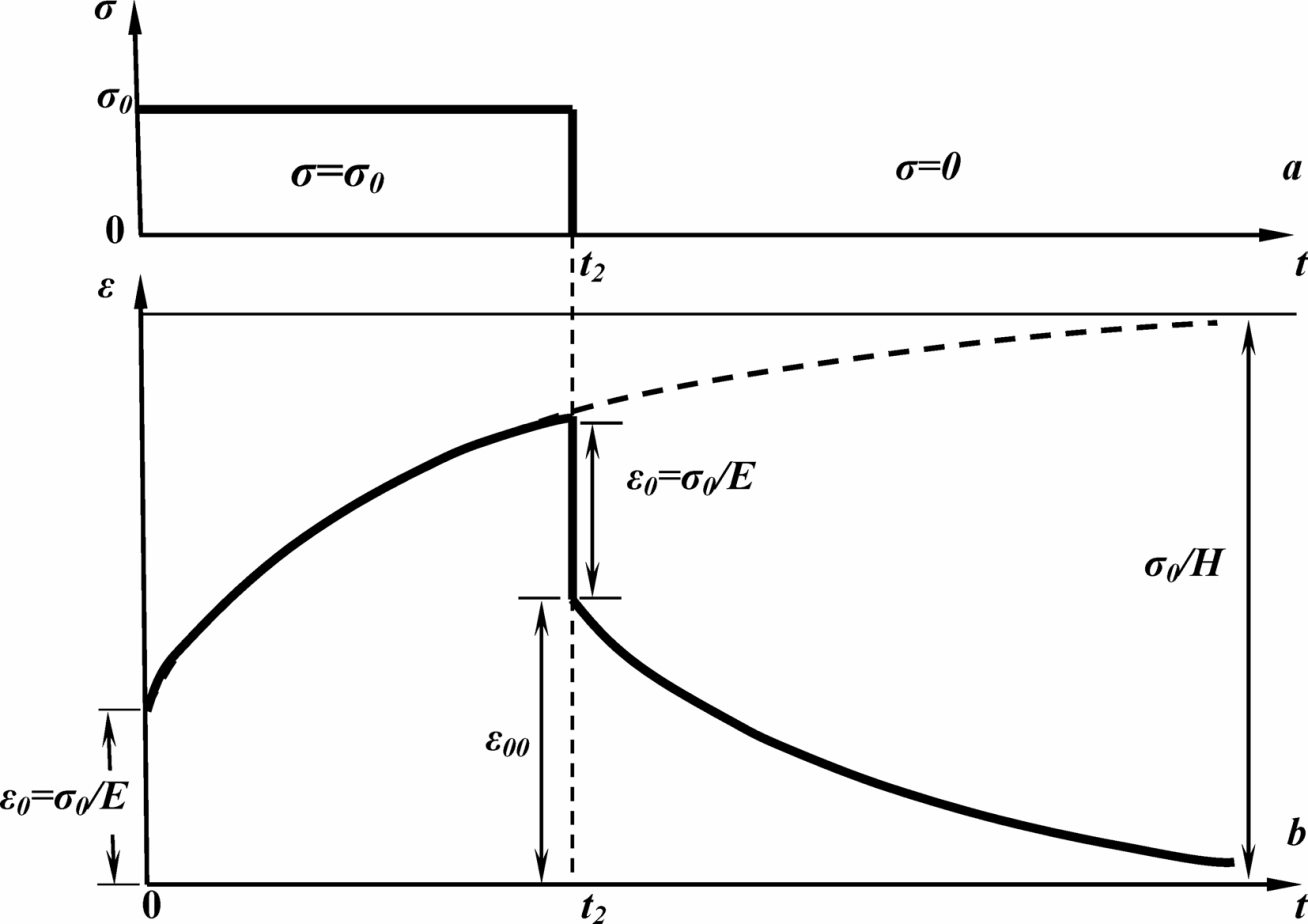
Mechanical stress applied to the sample versus time (***a***), and creep dynamics of the sample-relative deformation under conditions of stepwise loading/unloading (***b***).

From Eq. (18), we can say that the magnitude of the deformation *ε*(*t*) →0 at *t* → ꝏ. The *ε*(*t*) behavior under such loading is shown in Fig. 4.

## Results and discussion

**Fig. 5:**
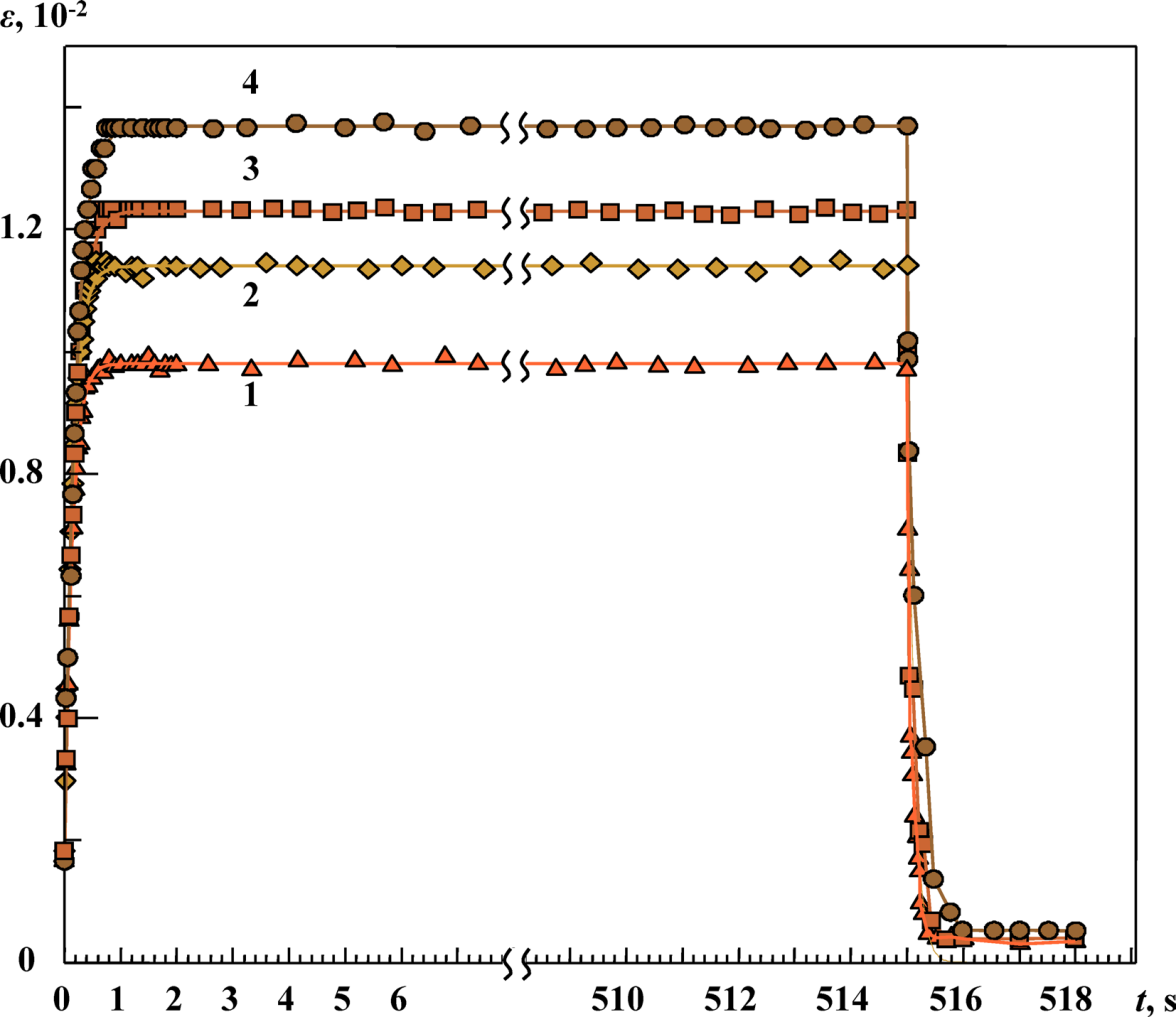
Dependence of creep curves *ε*(*t*) of aluminum alloy samples with ferromagnetic inclusions after application of load *σ*_0_ = 125 MPa (at time *t* = 0) and unloading (at time *t*_2_ = 515 s, *σ*(*t* > *t*_*2*_) = 0). Immediately before the creep tests, the samples had been exposed for 30 min at room temperature to a uniform magnetic field (MF) with induction *B*: **1** ― 0 T (witness sample), **2** ― 0.3 T, **3** ― 0.5 T, and **4** ― 0.75 T.

Typical experimental results for tensile loads *σ*_0_ = 125 N are shown in Fig. 5. We observed that preliminary exposure of the samples to a constant MF (with induction *B* = 0.75 T) for 30 min increased the deformation of the aluminum alloy. Thus, as seen from Fig. 5, at the tensile load *σ*_0_ under consideration, for the samples subjected to preliminary exposure to constant MF, the increase in absolute strain after the start of loading at the stage II of creep was as follows:

$$\varepsilon_{o\text{B}}-\varepsilon_o(\text{B} = 0.75 \text{T}) = 13.7\cdot10^{-3}-9.8\cdot10^{-3} = 3.9\cdot10^{-3}$$

$$\varepsilon_{o\text{B}}-\varepsilon_o(\text{B} = 0.3 \text{T}) = 11.4\cdot10^{-3}-9.8\cdot10^{-3} = 1.6\cdot10^{-3},$$

at 1 *s* < *t* < 530 s (Fig. 5). In addition, it was observed that, as previously noted in^22,23^, the creep rate of a polycrystalline Al alloy with Fe_x_Al_x−1_ inclusions, after exposing the samples to a constant MF, increased by 25%‒30%.

**Fig. 6:**
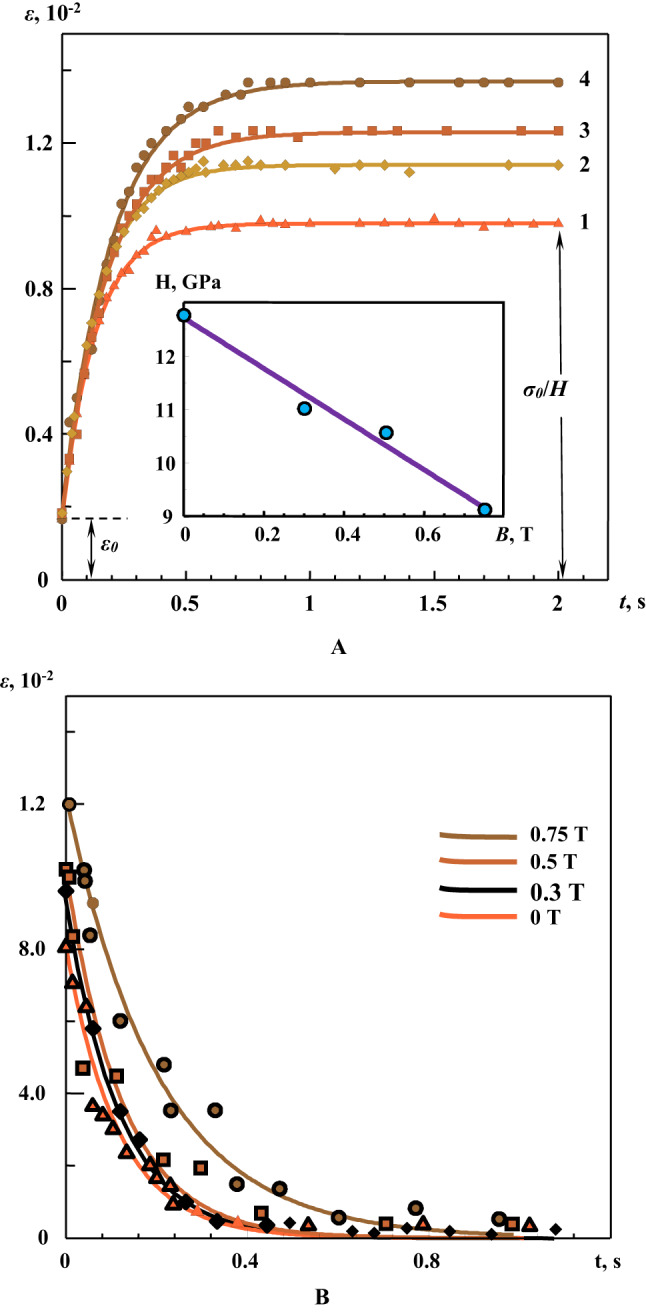
Family of creep curves ε(*t*) of aluminum alloy samples after application of a load (*σ* = *σ*_*0*_ = 125 MPa) at time *t* = 0 (**a**) and after removal of the load *σ*_*0*_ at time *t* = 0 *σ*(*t* > 0) = 0 (**b**) after exposing the samples to a constant MF with induction: **1** ― 0 Т (witness sample), **2** ― 0.3 Т, **3** ― 0.5 Т, and **4** ― 0.75 T. Solid lines indicate approximation according to Eq. (12). The approximation results are presented in Table 1. **Inset in** Fig. 6a. Dependence of modulus H on magnitude of the constant MF induction. Immediately before creep testing, the samples had been exposed to a constant MF for 30 min at room temperature.

Thus, in this series of experiments, the so-called short-term creep was implemented with characteristic relaxation times *n* ranging from milliseconds to seconds. The dynamics of the relative strain *ε*(*t*) after stepwise loading is shown in Fig. 6a. As expected, with stepwise loading of the sample with time, the deformation *ε*(*t*) increased and asymptotically approached $\frac{{{\sigma _0}}}{H}$ (Figs. 4 and 6a). In Fig. 6a, solid lines indicate the approximation results of a family of curves *ε*(*t*) after their preliminary exposure to a constant MF (with magnetic induction B = 0.3‒0.75 T). The approximation results are presented in Table 1. Clearly, all curves have been satisfactorily approximated via an exponential dependence in accordance with Eq. (12). The value of the modulus *E* was determined to be 72‒77 GPa based on experimental data and approximation results, which agrees well with the known literature data^29,30^. The relaxation time (*n* ~ 25 ms) and elastic modulus *H* were also determined, which exhibited a linear dependence on preliminary magnetic exposure (inset, Fig. 6a).

**Table 1:** Field dependence of mechanical characteristics of the samples under Stepwise loading/unloading, obtained via approximation of experimental data (*values directly obtained from experimental data).

No.	Parameter	Magnitude of magnetic field (MF) induction B, T			
		0	0.3	0.5	0.75
Step load, σ = 125 MPa $\varepsilon (t)=A\left( {1 - \left( {1 - B} \right){e^{ - Ct}}} \right),$ where $A=\frac{{{\sigma _0}}}{H},B=\frac{H}{E},C=\frac{H}{{nE}}$					
1	Coefficient *A*, 10^−2^	0.98	1.14	1.23	1.37
2	Coefficient *B*, 10^−1^	1.66	1.52	1.40	1.31
3	Coefficient *C*, s^−1^	7.41	6.63	5.47	4.98
4	Modulus *Е*, GPa	77	72	73	73
5	Modulus *Н*, GPa	12.8	11.0	10.6	9.1
6	Relaxation time *n*, ms	22	23	26	25
7	Paired correlation coefficient, *r*	0.998	0.997	0.996	0.992
8^*^	Relative strain *ε*_*0*_, 10^−3^	1.7	1.8	1,8	1,7
9^*^	Relative strain *ε*_*00*_, 10^−3^	8.1	9.6	10,2	12.0
10^*^	Modulus *Е = σ/ε*_*0*_, GPa	75	68	68	75
Stepped unloading, σ = 0 $\varepsilon (t)={\varepsilon _{00}}\exp \left({ - {C'}(t - {t_2})} \right)$, where ${C'}=\frac{H}{{{n'}E}}$					
11	Coefficient *C*^*’*^, s^−1^	8.83	8.4	8.08	4.87
12	Relaxation time *n*^*’*^, ms	19	18	18	26
13	Paired correlation coefficient, *r*^*’*^	0.967	0.954	0.942	0.967

As previously noted, the long-term elastic modulus *H* is a structure-sensitive parameter that indicates the impact of external influences on the mechanical properties of the aluminum alloys under study (including creep). Decrease in *H* with increasing *B* indicates changes in dynamics of the linear defects, which resulted in increase in creep. As discussed in the abovementioned text, the magnetostriction of inclusions (during preliminary exposure to MF) contributed to the emergence of additional mechanical stresses. Increase in *B* induced increase in local stresses at the interphase boundaries, at which the density of structural defects increased. Such changes in defect structure affected the subsequent deformation process, including transient processes during loading and stress relaxation during unloading.

For the considered aluminum alloy, strengthened via microscopic Fe-containing inclusions, the three-link Kelvin model was used (Fig. 2d). The model considered the elastic moduli of the matrix *E*_*1*_ and that of the inclusion *E*_*2*_. If the stress *σ*_*0*_ is specified as a step (Fig. 4, “instantaneous” loading), then the strain *ε*(*t*) tends to a constant value $\varepsilon (t)=\frac{{{\sigma _0}}}{H}$, and *H* in our model is calculated as follows:

18 $$H={\left( {\frac{1}{{{E_1}}}+\frac{1}{{{E_2}}}+...+\frac{1}{{{E_k}}}} \right)^{ - 1}},$$

where *E*_*k*_ is the modulus of elasticity of k-element.

Because the number of elements is *k* = 2 in the model under consideration, we have

19 $$H=\left( {\frac{{{E_1}{E_2}}}{{{E_1}+{E_2}}}} \right).$$

Owing to the preliminary exposure of the samples to an MF and the subsequent magnetostriction of inclusions, plastic deformation occurred in the border areas around the inclusions, which resulted in change in elastic modulus *E*_*1*_ and, accordingly, *H*.

In addition to the increase in plastic deformation of the material in the creep region after preliminary magnetic exposure, changes in the dynamics of deformation were registered at the stage of stress relaxation during unloading (Fig. 6b). Clearly, in contrast with the loading process, the dynamics of *ε*(*t*) are low-dependent on magnitude of magnetic induction, with the possible exception of the case with *B* = 0.75 T. Using the obtained values of *H* in the series 1 of experiments (during loading), the relaxation times were determined to be *n* ~ 19‒26 ms, which are consistent with those determined under stepwise loading (Table 1). In our previous work^24^, we determined the effect of pre-exposure of samples with iron inclusions on the changes in the nature of the formation of the zone of intense plastic deformation (“neck”). In this work, based on these data, we studied magnetically sensitive creep transients in structurally inhomogeneous aluminum alloys with iron-containing inclusions.

## Conclusions

In this study, we investigated the creep behavior of an aluminum alloy containing ferromagnetic inclusions (3–5 μm in size) during stages I and II, as well as the effect of preliminary exposure to a constant magnetic field with induction B < 0.75 T on this behavior. The experimental results demonstrated that the characteristic times of transient processes during stepwise loading and unloading were approximately 25 ms, corresponding to the short-term creep mode.

Based on the analytical solution for a rod with a rectangular cross-section subjected to stepwise loading and unloading, along with the experimental data, we determined the numerical values of the elastic modulus of the material and assessed the impact of preliminary magnetic exposure. The long-term elastic modulus *H* (i.e., the ratio of stress to relative deformation of a material after long-term application of constant load) decreased after preliminary magnetic exposure of the samples. This study emphasizes that the elastic modulus *H* is a structure-sensitive parameter that indicates the influence of external MFs on the creep dynamics of the structurally heterogeneous aluminum alloy under study. We believe that the decrease in *H* with increasing magnetic induction *B* is associated with the magnetostriction of inclusions (during preliminary exposure to MF). The increase in *B* increased the local stresses at the matrix–inclusion interfaces, where the dislocation density increased. These structural changes affected the subsequent deformation behavior of the alloy, including the transient creep response during loading and the stress relaxation observed during unloading.

## Data Availability

All research data are available from the corresponding author on reasonable request.
